# Current Knowledge on the Multifactorial Regulation of Corpora Lutea Lifespan: The Rabbit Model

**DOI:** 10.3390/ani11020296

**Published:** 2021-01-25

**Authors:** Massimo Zerani, Angela Polisca, Cristiano Boiti, Margherita Maranesi

**Affiliations:** Dipartimento di Medicina veterinaria, Università di Perugia, via San Costanzo 4, 06126 Perugia, Italy; massimo.zerani@unipg.it (M.Z.); boiti.cristiano@gmail.com (C.B.); margherita.maranesi@unipg.it (M.M.)

**Keywords:** rabbit, corpus luteum, reproduction

## Abstract

**Simple Summary:**

Corpora lutea (CL) are temporary endocrine structures that secrete progesterone, which is essential for maintaining a healthy pregnancy. A variety of regulatory factors come into play in modulating the functional lifespan of CL, with luteotropic and luteolytic effects. Many aspects of luteal phase physiology have been clarified, yet many others have not yet been determined, including the molecular and/or cellular mechanisms that maintain the CL from the beginning of luteolysis during early CL development. This paper summarizes our current knowledge of the endocrine and cellular mechanisms involved in multifactorial CL lifespan regulation, using the pseudopregnant rabbit model.

**Abstract:**

Our research group studied the biological regulatory mechanisms of the corpora lutea (CL), paying particular attention to the pseudopregnant rabbit model, which has the advantage that the relative luteal age following ovulation is induced by the gonadotrophin-releasing hormone (GnRH). CL are temporary endocrine structures that secrete progesterone, which is essential for maintaining a healthy pregnancy. It is now clear that, besides the classical regulatory mechanism exerted by prostaglandin E2 (luteotropic) and prostaglandin F2α (luteolytic), a considerable number of other effectors assist in the regulation of CL. The aim of this paper is to summarize our current knowledge of the multifactorial mechanisms regulating CL lifespan in rabbits. Given the essential role of CL in reproductive success, a deeper understanding of the regulatory mechanisms will provide us with valuable insights on various reproductive issues that hinder fertility in this and other mammalian species, allowing to overcome the challenges for new and more efficient breeding strategies.

## 1. Introduction

Corpora lutea (CL) are temporary endocrine structures that secrete progesterone, which is essential for a healthy pregnancy in most species. In rabbits, the CL develop rapidly following ovulation and reach their maximum size and functional capacity within nine to ten days. This process shows the intense angiogenesis and active granulosa or theca cell luteinization of preovulatory follicles, due to the effects of several local angiogenic growth factors, gonadotropins and other hormones [1,2]. In pregnant rabbits, the CL lifespan lasts for about 30 days [3]; however, if pregnancy does not occur, the lifespan of the CL is much shorter, and luteal regression starts around day 12 and ends 16 days after ovulation when the peripheral plasma progesterone concentrations drop to the baseline values [4,5]. Therefore, the absence of embryonic signals or the end of gestation activates luteolysis, a comprehensive regressive process that leads to total functional and structural CL demise, in which prostaglandin (PG) F2 α (PGF2α) plays a central role [6].

Many regulatory factors, including cytokines, growth factors, prostaglandin E2 (PGE2) and PGF2α released by different CL cell types, including endothelial and local immune cells and fibroblasts, as well as progesterone and 17β-estradiol released by luteal and follicular cells and hormones, control the functional lifespan of the CL, with luteotrophic and luteolytic effects [7]. However, the overall balance between these contrasting actions varies considerably with the age of the CL and/or in the presence/absence of an embryo [8]. Many facets of luteal physiology have been clarified, but others are still poorly understood, including the molecular and/or cellular mechanisms that protect the CL from luteolysis from the early luteal phase. Moreover, the mechanisms that are induced by the administration of exogenous PGF2α have been extensively investigated in rabbits [9,10,11] in order to evaluate PG paracrine and/or autocrine functions and other possible regulators that switch on (luteotropic)/off (luteolyic) progesterone production by the CL at a specific stage of its life cycle. However, there are few data on the mechanisms that protect the developing CL from functional luteolysis in the early luteal phase, which starts on day six of a pseudopregnancy, when the luteal cells acquire the ability to respond to the luteolytic effects of exogenous PGF2α (luteolytic capacity) [9]. Luteolysis is a key event in reproduction for spontaneously ovulating species, as well as for rabbits, whose mating activity triggers a neuroendocrine reflex, which, combined with GnRH or exogenous human chorionic gonadotropin (hCG) exogenous administration, induces ovulation [12,13].

This paper provides a summary of our current knowledge on the endocrine and cellular mechanisms of multifactorial CL lifespan regulation, acquired using the pseudopregnant rabbit model, which was able to determine the relative luteal phase following GnRH-induced ovulation. Most of the mechanisms described in this review were observed during our studies on the progressive age-dependent response of the CL to PGF2α conducted over a 20-year period [9]. A better understanding of these mechanisms may provide us with valuable insights in the challenge to find more efficient breeding strategies for rabbits, as well as for other species.

## 2. Prostaglandins

Prostaglandins (PGs) play a key regulatory role in CL function and the lifespan: PGF2α is the main luteolytic agent produced by the uterine endometrium of numerous mammals, including rabbits, but not by primates [14,15,16], while PGE2 plays a crucial luteoprotective role, with luteotrophic and/or antiluteolytic effects [6]. In some species, PGF2α and PGE2 are produced by the CL [17,18,19,20,21].

An essential step in PG biosynthesis is the cyclooxygenase (COX) 1 (COX1) and/or COX2 enzymatic conversion of arachidonic acid (AA)—produced by phospholipase A2 (PLA2) activity—into PGH2 [22,23,24]. This latter PG is then transformed into four structurally active PGs (PGE2, PGF2α, PGD2 and PGI2) by specific PG synthases [25]. PGF2α biosynthesis is particular, since three specific ketoreductases catalyze this PG from PGH2, PGD2 or PGE2, respectively [26]. PGE2-9-ketoreductase (PGE2-9-K) is present in the rabbit ovary [27] and CL [28]. This ketoreductase also converts progesterone into its inactive metabolite through its 20α-hydroxysteroid dehydrogenase (HSD) catalytic activity.

We previously reported [21] that, in rabbits, intra-luteal PGF2α activates luteolysis with an auto-amplification loop: during the mid- and late-luteal phases, it activates COX2 and PGE2-9-K; the former converts AA into PGH2, which is then transformed into PGF2α and PGE2, while the latter is converted into PGF2α through PGE2-9-K activation. Moreover, this enzyme significantly reduces PGF2α-induced progesterone through its 20α-hydroxysteroid dehydrogenase (20α-HSD) activity that converts progesterone into 20α-OH-progesterone. Late-luteal phase PGE2 production plays another essential role: PGE2-9-K enzymatic activity make this PG the main source of PGF2α synthesis.

**Figure 1:**
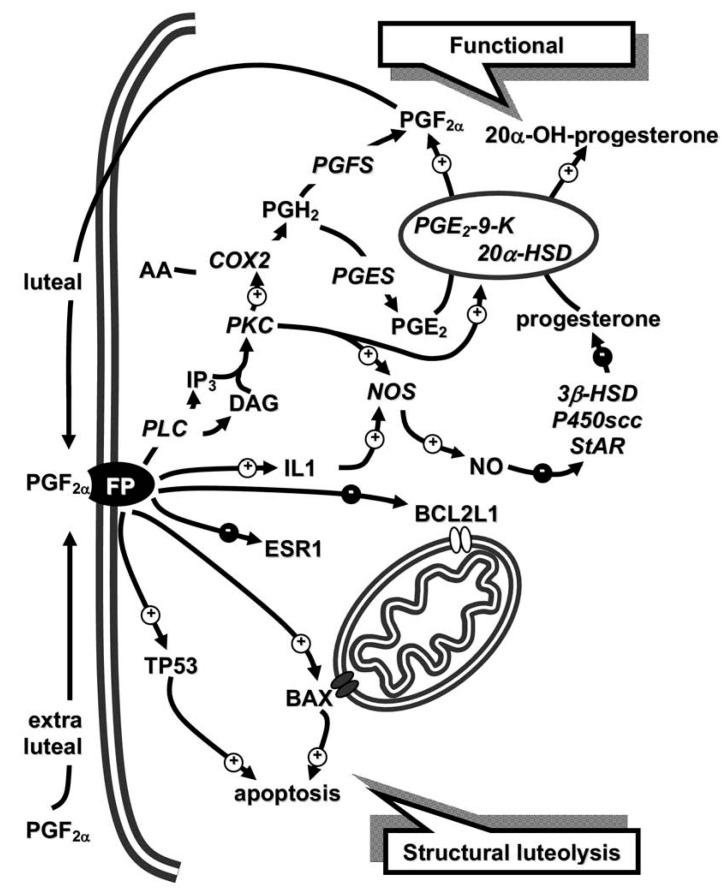
Schematic model reporting the functional (upper) and structural (lower) luteolytic pathways induced by prostaglandin F2 α (PGF2α) in rabbit mid-corpora lutea (CL) (day 9 of pseudopregnancy). Since prostaglandin E2 (PGE2)-9-K and 20α-hydroxysteroid dehydrogenase (HSD) represent two different activities of a single enzyme, they are joined. Figure from the study by Maranesi et al. 2010 [31]. For acronyms, see the list of abbreviations in the text.

Arosh et al. [29] suggested that CL PG biosynthesis is mainly directed toward PGE2 production rather than PGF2α. In fact, PGH2 conversion into PGE2 (PGE synthase) is 150-fold higher than that of PGH2 into PGF2α (PGF synthase) [30]. These results [29,30], combined with our data [21,31], allow us to hypothesize [31] that rabbit CL in the early and mid-luteal phases use the same cellular enzymatic pathways (PLA2/AA/COX2/PGH2/PGE synthase/PGE2) to produce an initial PGE2 amount, while the final luteal production of PGE2 (early CL) or PGF2α (mid-CL) is regulated by PGE2-9-K inactivation or activation, respectively (Figure 1, upper, functional luteolysis).

Several studies have investigated the possible factors involved in PGF2α-induced luteolytic capacity during the mid-luteal phase [7,9,32,33,34,35,36]. Interleukin 1 (IL1), with other cytokines that are normally present in rabbit luteal cells [32,33], are locally involved in the CL function control leading to apoptosis as proinflammatory mediators [34]. Moreover, locally acting hormones and pro- and antiapoptotic intra-luteal factors may interact dynamically. 17β-Estradiol is one of the main luteotropic effectors, since its absence leads to luteolysis through apoptosis activation [7]. Nitric oxide synthase (NOS) and its product nitric oxide (NO) are also known to have pro- and antiapoptotic properties that modulate various intracellular pathways—in particular B-cell CLL/lymphoma 2 (BCL2)-like 1 (BCL2L1) and tumor protein p53 (TP53) proteins [35]. In rabbits, NOS luteal inhibition favors apoptosis [36].

Our study [31] on the key protein-encoding genes involved in apoptotic mechanism control revealed that PGF2α induces luteolysis in luteal cells with an acquired luteolytic capacity through the upregulation of luteal IL1B and TP53 gene transcripts and the downregulation of the estrogen receptor 1 (ESR1) and BCL2L1 receptors. This PGF2α-induced CL regression seems to be the result of two distinct mechanisms: the steroidogenic pathway, by ESR1 downregulation, and the apoptotic pathway, by the dynamic changes of the TP53 and BCL2L1 proteins and gene transcripts (Figure 1, lower, structural luteolysis). Finally, aglepristone (RU534), an antiprogestinic, increases progesterone release in rabbit mid- and late-CL, whereas this antiprogestinic reduces PGF2α and enhances PGE2 only during the late-luteal stage [37].

## 3. Nitric Oxide

Nitric oxide is a potent vasodilator factor involved in several biological processes, such as neurotransmissions and cytotoxicity, under both physiological and pathological conditions [38,39]. NO is produced by the enzymatic action of NOS, which converts L-arginine into NO and L-citrulline. There are three forms of NOS: two constitutive Ca^2+^-dependent forms neuronal NOS (nNOS) and endothelial NOS (eNOS) and an inducible Ca^2+^-independent form (iNOS) [38,40]. With the exception of neuronal and endothelial cells, constitutive eNOS and nNOS are normally expressed in various cell types and produce low levels of NO. Contrastingly, the inducible form only produces large quantities of NO when the expression is activated [38,40]. NOS is present in both ovarian stroma and follicular granulosa cells of several mammalian species, including rabbit ovaries, where it regulates steroidogenesis [17,41,42,43,44]. The NO/NOS system present in rabbit, rat and mare ovaries is also involved in ovulation [43,44,45,46,47,48,49]. All of these studies suggest that NO regulates the key mechanisms of ovarian physiology.

In rabbits, NO has a direct antisteroidogenic effect at the luteal level. Numerous in vivo and in vitro experiments have found that NO and NOS are the main targets of PGF2α and effectors of PGF2α-induced luteolysis in competent CL [10,11,17,18,33,50]. Ovarian NO is known to be a mediator of the luteolytic action induced by PGF2α in rabbits and other mammalian species [17,51,52,53,54,55]. Ovarian NO might also control the CL lifespan by regulating 17β-estradiol and progesterone concentrations. However, in contrast to earlier findings in rat and human in vitro cultured CL [41,56], NO did not affect the total androgens and 17β-estradiol production in rabbit CL [17]. Contrastingly, in rabbit CL cultured in vitro, the NO donor, sodium nitroprusside, greatly reduced progesterone secretion in all luteal developmental stages [17]. Luteal NOS activity decreases between the early-to mid-luteal phases with elevated steroidogenesis levels [17,57], which increase again in late-CL when the progesterone levels drop and natural luteolysis initiates [5,57].

## 4. Leptin

Leptin is a cytokine secreted mainly by adipocytes and encoded by the obese gene [58]. Leptin regulates the hypothalamic centers of satiety and energy metabolism through the modulation of various neurotransmitters [59,60].

The leptin receptor (ObR) has six isoforms (a–f) resulting from mRNA splice variants [61,62]. ObRa–d and ObRf have identical extracellular and transmembrane domains [62,63]. A long intracellular domain of ObRb activates the Janus kinase (JAK)/signal transducer with the subsequent signal transducer and activator of transcription (STAT) phosphorylation [64]. Contrastingly, the short intracellular domain of ObRa, ObRc,d and ObRf activates the mitogen-activated protein kinase (MAPK) pathway [61,65].

Several studies have found that various key mammalian reproductive processes are modulated by leptin [66], including steroidogenesis [67,68], ovulation [69,70], pregnancy [71,72] and menstrual cycles [73,74]. Moreover, leptin is the crucial link between adipose tissue and the reproductive system, since it provides information on whether energy reserves are adequate for normal reproductive function [75].

Leptin receptors are present in several tissues of the hypothalamic–pituitary–gonadal (HPG) axis and in pituitary [76], granulosa, theca and interstitial ovary cells [77]. Various studies have reported that leptin directly inhibits steroidogenesis in intracellular signaling pathways in theca, granulosa and luteinized granulosa cells of rodents, bovines and primates [67,68,77,78,79].

**Figure 2:**
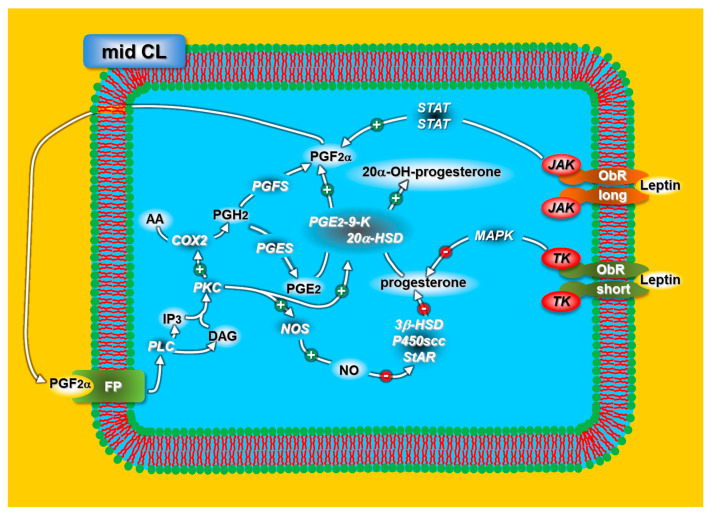
Schematic representation of the leptin mechanisms regulating progesterone release in rabbit mid-CL. For acronyms, see the list of abbreviations in the text.

Our studies on rabbit CL [80] show that leptin affects progesterone and PGF2α release with different intracellular signaling pathways through different receptors (long ObR and short ObR). More specifically, leptin inhibits progesterone release through the MAPK cascade (short ObR) and stimulates PGF2α release through the JAK pathway (long ObR) (Figure 2).

## 5. Gonadotropin-Releasing Hormone (GnRH)

Gonadotropin-Releasing Hormone (GnRH) is a hypothalamic-releasing decapeptide and a key regulator of the mammalian reproductive system. GnRH regulatory action on the reproductive functions is exerted largely via luteinizing hormone (LH) and follicle-stimulating hormone (FSH) secretion, which also affect steroidogenesis and germ cell development [81]. Although the hypothalamus and pituitary gland are the main GnRH synthesis and action sites, several studies have reported an extra-hypothalamic presence of GnRH and its cognate receptor (GnRHR) in numerous peripheral tissues, including reproductive organs such as the gonads, prostate, uterine tube, placenta and mammary glands [82]. Previous studies have highlighted that GnRH regulates the ovarian steroid hormones [82]. In rabbit CL, GnRH administration was found to be associated with CL regression with decreased levels of serum progesterone [83]. Contrastingly, no GnRH effects were observed on ovarian tissue steroid production by other authors [84].

**Figure 3:**
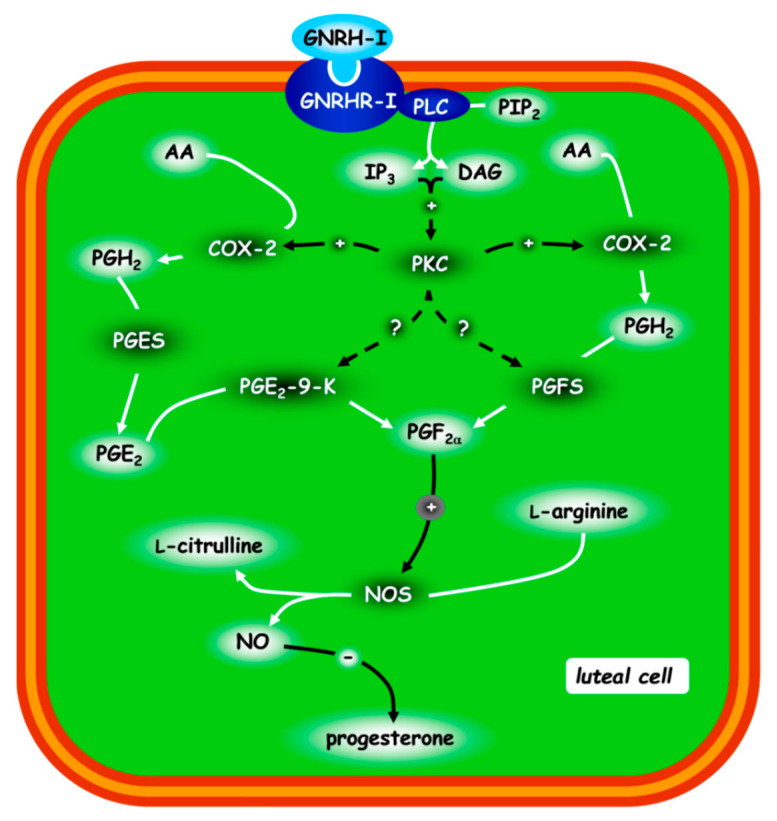
Schematic representation of the post-receptorial mechanism of GnRH-I regulating the progesterone release in rabbit CL. The other possible protein kinase C (PKC) targets are represented by hatched lines. Figure from the study by Zerani et al. 2010 [85]. For acronyms, see the list of abbreviations in the text.

The studies conducted in our laboratory [85] highlighted that the autocrine, paracrine and/or endocrine roles of GnRH type I (GnRH-I) directly diminished the progesterone secretion in rabbit CL that had acquired luteolytic competence (Figure 3): GnRH-I acts via GnRHR-I by activating phospholipase C (PLC) and stimulating the inositol trisphosphate (IP3) and diacylglycerol (DAG) pathways. Through the activation of protein kinase C (PKC), these two intracellular messengers stimulate COX2 activity and PGF2α release. This PG induces (via paracrine, autocrine and/or intracrine mechanisms) an increase in NOS activity and NO levels [11], which downregulates the progesterone levels [18,31] (Figure 1, upper, functional luteolysis).

## 6. Endothelin 1

Endothelin 1 (ET1), a 21-amino acid peptide, is a potent vasoconstrictor secreted by vascular endothelial cells [86,87]. Many tissues other than the vascular endothelium are known to express ET1, including follicular granulosa cells [88,89,90,91,92].

In rabbit CL, ET1 receptors are expressed in the vascular compartments and luteal cells, thus evidencing that the ET1 system is related to ovarian blood flow and steroid hormone production [91,92]. Moreover, ET1-induced luteolysis in rabbits on day nine of the pseudopregnancies was prevented by administering captopril, the angiotensin-converting enzyme inhibitor (ACE). It is important to note that PGF2α-induced luteolysis was not influenced by captopril. These findings indicate that the cascade mechanism triggered by PGF2α does not require the renin–angiotensin system for inducing luteolysis in rabbits [92], which is in good agreement with the data obtained for cows [93]. Strict cooperation between endothelin and NO is required for endothelial cell migration and angiogenesis [94]. ET1 was found to stimulate endothelial NOS under different physio-pathological conditions [95], while NO/NOS is a recognized system involved in both PGF2α [11] and ET1 [96]-induced luteal regression.

## 7. Adrenocorticotropic Hormone

Adrenocorticotropic hormone (ACTH) is a major component of the hypothalamic–pituitary–adrenal (HPA) axis, which is synthesized and secreted by the anterior pituitary gland in response to stress. This response is activated by the hypothalamic corticotropin-releasing hormone (CRH), which stimulates pituitary ACTH release, with subsequent glucocorticoid secretion from the adrenal glands.

There is strong evidence that female reproduction can be impaired by stress [97]. In fact, CRH, ACTH and glucocorticoid negatively affect fertility by targeting the hypothalamic GnRH neurons [98], as well as pituitary LH and/or FSH production and sex steroid synthesis by ovarian follicles and CL. However, the mechanisms by which hormones released during stress may inhibit reproductive mechanisms have yet to be clarified; however, any direct action of ACTH on ovarian functions requires the activation of melanocortin receptor 2 (MC2R) [99], while any indirect action requires glucocorticoid receptor (GR) activation.

**Figure 4:**
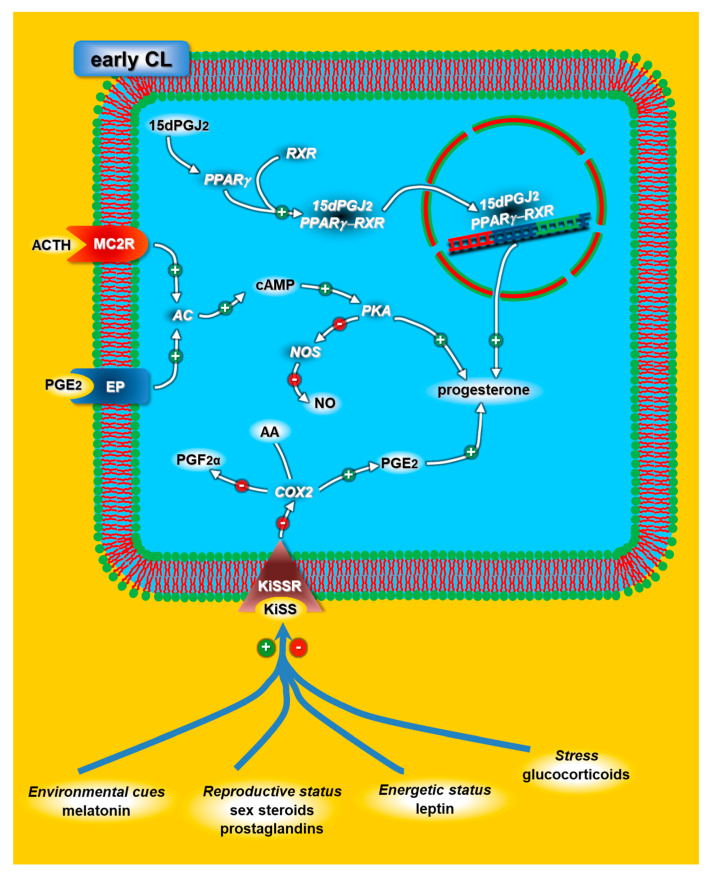
Schematic diagram of the adrenocorticotropic hormone (ACTH), kisspeptins (KiSS) and peroxisome proliferator-activated receptor (PPAR) mechanisms modulating progesterone release in early rabbit CL. The effectors that could directly modulate the KiSS/KiSSR (receptor) system at the CL level are represented by blue lines. For acronyms, see the list of abbreviations in the text.

The presence of ACTH and glucocorticoid receptors in the luteal cells of rabbit CL [100] supports the hypothesis that ACTH affects ovarian functions both directly and indirectly. During the early and mid-luteal phases (days four and nine of the pseudopregnancies), ACTH increased the in vitro progesterone and PGE2 releases but reduced the PGF2α release. Contrastingly, ACTH increased the in vivo plasmatic cortisol levels within four hours, while the progesterone levels dropped 24 h later and for the following 48 h. Daily injections of ACTH did not affect the progesterone profile following ovulation. Taken together, these findings indicate that ACTH directly induces the upregulation of luteal progesterone synthesis through MC2R (Figure 4), while it indirectly blocks CL functions through the cortisol/GR system.

## 8. Immunity Mediators

It is now widely accepted that luteolysis is an event mediated by immune effectors in rabbits and other species, as demonstrated by the presence of immune cells during spontaneous luteal regression [32]. Luteal immune cells are key modulators of CL activity, affecting the luteal, endothelial and stromal cells through several cytokines, including IL1, tumor necrosis factor (TNF)α, monocyte chemoattractant protein-1 (MCP1) and interleukin 2 (IL2) [33,101,102]. In rabbits, during spontaneous luteolysis, the expression levels of MCP1 and IL1β increased on day 15 of the pseudopregnancies [33]. These findings show the greater influx of macrophages and immune cells observed during luteal regression [103]. The IL2 transcript increases earlier (day 13 of the pseudopregnancies) than the other cytokines [33]; in fact, T lymphocytes were detected in rabbit CL before the macrophages [103].

The IL-1 cytokine is present in the ovaries of various species, including rabbits [104,105]. IL1β has various effects on the ovaries [106]: it inhibits progesterone production, increases PG synthesis and PGF2 receptor expression, it inhibits COX2 mRNA degradation [107], enhances NO production and induces the activation of constitutive and inducible NOS [108].

Our studies report [21] that injecting pseudopregnant rabbits with PGF2α markedly upregulated COX2 and IL1β mRNA expression and increased PGF2α release and COX2 activity only in CL with acquired luteolytic capacity [31]. These data suggest that IL1β enhances intra-luteal PGF2α synthesis by upregulating the luteal function of COX2 and NOS, thus promoting functional regression in luteal cells that have achieved luteolytic capacity.

## 9. Peroxisome Proliferator-Activated Receptor

The peroxisome proliferator-activated receptors (PPARs) include a family of three (a, d and c) nuclear receptor/transcription factors, which regulate steroidogenesis, angiogenesis, tissue remodeling, cell cycle and apoptosis [109], which are all essential processes for normal ovarian function [110]. All three PPARs have been detected in the ovaries of numerous species [111], including rats [110,112], mice [113], pigs [114], sheeps [115], cows [116,117,118], rabbits [119] and humans [120,121].

Komar [110] reported that PPARc activation affected the progesterone synthesis in ovarian cells. In particular, an endogenous activator of PPARc 15d-PGJ2 inhibited both the basal and gonadotropin-induced production of progesterone in human granulosa cells [122], while 15d-PGJ2 and ciglitazone, a synthetic PPARc activator, increased progesterone production by granulosa cells in equine chorionic gonadotropin (eCG)-primed immature rats [123]. PPARc activation by 15d-PGJ2, ciglitazone or another synthetic activator, troglitazone, also increased progesterone release by porcine theca and bovine luteal cells [114,124]. Taken together, these findings indicate that the cell type, stage of cell differentiation, stage of the ovarian cycle and/or animal species influence the effects of PPARc on progesterone production [110].

Our study [125,126] suggests that PPARc may play a luteotropic role in rabbit CL through a mechanism that upregulates 3β-hydroxysteroid dehydrogenase (3β-HSD) and increases progesterone while it downregulates PGF2α and its correlated enzyme COX2 [21] (Figure 4). Moreover, the significant decrease in PPARc in the luteal cell nucleus during the late-luteal stage supports the aforementioned mechanism, thus suggesting that this reduction may be required for luteolysis to take place.

## 10. Dopamine

The catecholamine dopamine (DA) is a neurotransmitter widely distributed in the brain and in various peripheral organs of numerous species [127]. DA exerts its physiological actions by binding to specific receptors (DR). In mammals, there are five dopamine receptor subtypes, which are grouped into the D1R-like and D2R-like receptor superfamilies [127,128].

D1R-like receptors stimulate the production of the second messenger cyclic adenosine monophosphate (cAMP); contrastingly, D2R-like receptors inhibit cAMP synthesis, which decreases the protein kinase A (PKA) activity [128]. In mammals, dopamine receptors are widely expressed in many organs and tissues, including the reproductive system [128]. D1R has been detected in the luteal cells of humans [129,130], horses [131], rats [132], cows [118] and rabbits [133], suggesting that DA might be directly involved in the physiological pathways regulating the CL function.

**Figure 5:**
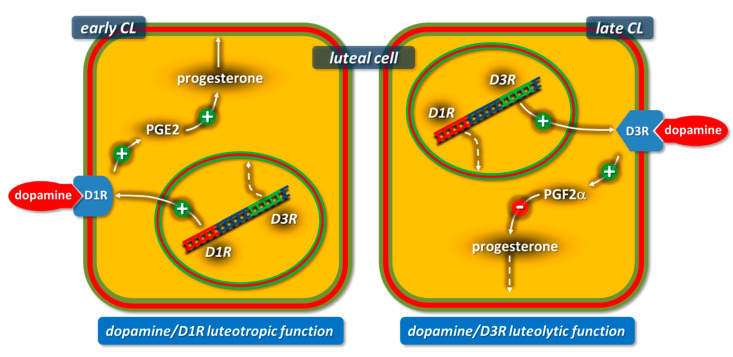
Schematic representation of the dopamine receptor-dependent mechanism modulating progesterone production in early (Day 4) and late (Day 9) rabbit CL. D1R: dopamine receptor subtype 1 (cAMP increase) and D3R: dopamine receptor subtype 3 (cAMP decrease). Italic: D1R and D3R genes. Figure from the study by Parillo et al. 2014 [132].

Our studies [133] provide evidence that CL produce DA and that the DA/D1R-D3R system regulates the CL lifespan by exerting either luteotrophic or luteolytic actions depending on the luteal stage. In fact, the DA/D1R-D3R system stimulated PGE2 and progesterone synthesis by early CL, while it increased PGF2α production and decreased progesterone production by mid- and late-CL (Figure 5).

A multi-synaptic neural pathway connects the ovaries to the central nervous system in mammals [134]. Moreover, the ovarian interstitial stroma is composed of many different cell types, including neuron-like or neuroendocrine cells [135]. These data suggest that extrinsic and intrinsic neurons are another paracrine source of DA that can bind its cognate receptors D1R and D3R in the CL, thus supporting the hypothesis that the DA/DR system plays a physiological role in regulating the CL lifespan and functions.

## 11. Kisspeptin

The hypothalamic neuropeptide kisspeptins (KiSS) are greatly involved in mammalian reproduction. In fact, they regulate the synthesis and production of GnRH that are required to initiate puberty and sustain normal reproductive function [136].

KiSS and its receptor KiSS1R are expressed in various ovarian structures, including the CL of several mammalian species [137,138,139], supporting the hypothesis that these neuropeptides can regulate the CL lifespan by modulating the steroidogenic enzymes controlling progesterone synthesis. Moreover, Laoharatchatathanin et al. [140] suggested that KiSS is involved in the luteinization of rat granulosa cells.

Based on data obtained in our laboratory [141], we hypothesize that, besides the well-known hypothalamic mechanism, the KiSS/KiSS1R system may also directly control the rabbit CL lifespan via local mechanisms. In fact, KiSS was found to exert a luteotrophic action by increasing luteal progesterone synthesis, likely through autocrine and/or paracrine mechanisms that simultaneously reduce PGF2α production and increase PGE2 production by blocking COX2 activity (Figure 4). The lack of KiSS1R expression in late-CL suggests that the functional activity of the KiSS/KiSS1R system is mainly regulated by the gene and/or protein expression of the receptor.

Interestingly, there is sufficient evidence to suggest that the hypothalamic KiSS-1 gene expression is regulated by several factors, including melatonin, gonadal steroids and leptin, which convey environmental cues and reproductive and metabolic conditions, respectively [142,143]. The theory that these factors could modulate the luteal KiSS/KiSS1R system cannot be ruled out (Figure 4).

## 12. Nerve Growth Factor

The nerve growth factor (NGF), together with brain-derived growth factor and other neurotrophins, belong to the neurotrophin family [144]. These neurotrophins maintain normal physiological functions in the central and peripheral nervous systems, including neural development, differentiation and synaptic plasticity [145,146]. NGF and its receptors neurotrophic receptor tyrosine kinase 1 (NTRK1) and nerve growth factor receptor (NGFR) have been found in rabbit ovaries [147,148]. In particular, our studies [149] have evidenced that NGF from seminal plasma supports the neuroendocrine ovulatory reflex induced by mating and/or vaginal stimulation through a novel mechanism exerted on the uterus and/or cervix.

Although there is sufficient experimental evidence suggesting that seminal plasma NGF is able to induce ovulation in rabbits [147], its potential role in regulating the CL lifespan has not yet been thoroughly explored. To date, we only know that NGF and its cognate receptor NTRK1 are expressed in rabbit CL at various stages of a pseudopregnancy [149]. Contrastingly, using purified NGF obtained from seminal plasma, Silva et al. [150,151] observed that, in llamas, CL increased vascularization, upregulated cytochrome P450, family 11, subfamily A, member 1/P450 side chain cleavage and steroidogenic acute regulatory protein transcripts and increased progesterone secretion. All of these findings support the hypothesis that NGF positively affects CL development. Tribulo et al. [152] and Stewart et al. [153] obtained similar results in heifers; however, no luteotrophic effect was observed in alpaca CL using recombinant human NGF [154,155].

## 13. Conclusions

In conclusion, it is now well-documented that the progressive acquisition of luteolytic competence by rabbit CL is not only due to their increased sensitivity to PGF2 induced by the upregulation of PGF2α and its receptors and to the decrease of the luteotropic factors (E2, PGE2 and ACTH), but it is also caused by several antisteroidogenic factors. These include, among others, GnRH, ET1 and leptin, which influence the inflammatory, vascular and apoptotic processes involved in the luteolytic process through interaction with PGF2α and the NO/NOS system. During PGF2α-induced CL regression with luteolytic competence, all these factors concomitantly induce the upregulation of NOS, COX2 and PGE2-9-K activities and gene transcripts for ETI, COX2, IL1B and TP53, as well as the downregulation of several other transcripts, including ESR1 and BCLXL. Therefore, the luteolytic effect of PGF2α is the result of its influence on distinct processes involving the regulation of vasoactive peptides, steroidogenic pathways and apoptotic pathways. However, despite the increased knowledge on the physiology of rabbit CL, it is recommended that further research should be undertaken in the near future by a younger generation of researchers who will be able to apply these new discoveries in the challenge for new rabbit breeding strategies.

## Abbreviations

3β-HSD	3β-hydroxysteroid dehydrogenase
15d-PGJ2	15-deoxy-Δ12,14-prostaglandin J2
20α-HSD	20α-hydroxysteroid dehydrogenase
AA	arachidonic acid
ACE	angiotensin converting enzyme
ACTH	adrenocorticotropic hormone
BAX	BCL2-associated X protein
BCL2L1	B-cell CLL/lymphoma 2 (BCL2)-like 1
cAMP	cyclic adenosine monophosphate
CL	corpora lutea; COX1
COX1	cyclooxygenase 1
COX2	cyclooxygenase 2
CRH	corticotropin-releasing hormone
DA	dopamine
DAG	diacylglycerol
DR	dopamine receptor
eCG	equine chorionic gonadotropin
eNOS	endothelial NOS
ESR1	estrogen receptor subtype-1
ET1	endothelin 1
GnRH	gonadotropin-releasing hormone
GnRH-I	gonadotropin-releasing hormone type I
GnRHR	gonadotropin-releasing hormone receptor
GR	glucocorticoid receptor
hCG	human chorionic gonadotropin
HPA	hypothalamic-pituitary-adrenal
HPG	hypothalamic–pituitary–gonadal
IL1B	interleukin 1 Beta
iNOS	inducible NOS
IP3	inositol trisphosphate
JAK	Janus kinase
KiSS	kisspeptin
KiSSR	kisspeptin receptor
MAPK	mitogen-activated protein kinase
MC2R	melanocortin receptor type 2
MCP1	monocyte chemoattractant protein-1
nNOS	neuronal NOS
NGF	nerve growth factor
NGFR	nerve growth factor receptor
NO	nitric oxide
NOS	nitric oxide synthase
NTRK1	neurotrophic receptor tyrosine kinase 1
ObR	leptin (obesity) receptor
P450scc	P450 side-chain cleavage
PG	prostaglandin
PGD2	prostaglandin D2
PGE2-9-K	PGE2-9-ketoreductase
PGE2	prostaglandin E2
PGF2α	prostaglandin F2α
PGH2	prostaglandin H2
PGI2	prostaglandin I2
PKA	protein kinase A
PKC	protein kinase C
PLA2	phospholipase A2
PLC	phospholipase C
PPAR	peroxisome proliferator-activated receptor
RXR	retinoid X receptor
StAR	steroidogenic acute regulatory protein
STAT	signal transducer and activator of transcription
TK	tyrosine kinase
TNFα	tumor necrosis factor α
TP53	tumor protein p53
